# Role of youth in combating climate change

**DOI:** 10.4103/0019-5278.55130

**Published:** 2009-08

**Authors:** Harshal T. Pandve, Poonam R. Deshmukh, Rahul T. Pandve, Neha R. Patil

**Affiliations:** 1 Department of Community Medicine, Smt. Kashibai Navale Medical College, Narhe, Pune-411 041, India; 2 Department of Epidemiology, School of Public Health, University of Alabama, Birmingham, Ryals Public Health Building, Suite 430, Birmingham, AL, 35294, USA

Climate change is one of the most critical global challenges of our times. Recent events have emphatically demonstrated our growing vulnerability to climate change. Climate change impacts range from affecting agriculture, further endangering food security, to sea-level rise and the accelerated erosion of coastal zones increasing the intensity of natural disasters, species extinction, and spread of vector-borne diseases. This issue is of immense importance for every global citizen. Hence it requires an initiative against it globally.[1]

Youth play a crucial role in combating climate change. A questionnaire-based pilot survey was conducted in Pune city of Maharashtra state, Indiam to assess awareness about climate change among the college going youth. Amongst 201 respondents 66.2% were males and 33.8% were females studying in various faculties or courses. About 98.5% respondents said global climate is changing, 95.5% of the respondents also commented that human activities contribute to climate change. The study also assessed awareness regarding major international organizations and panels working on global climate change and its effects. Only 45.3% of the respondents knew about the United Nations Framework Convention on Climate Change (UNFCC) and the Kyoto Protocol while 45.8% were aware of the Inter-governmental Panel on Climate Change (IPCC) which conducts scientific analysis of climate change, global warming and its impacts. About 54.5% of the respondents believed that youth could play a major role in combating climate change.

As per the 60th annual DPI/NGO conference organized by the United Nations Department of Public Information (DPI) in collaboration with the NGO/DPI, an executive committee meet on “Climate Change: How It Impacts Us All” was held from September 5 to 7, 2007, at the United Nations Headquarters. It stressed the role of youth, the next generation which inhabits the Earth and inherits the responsibility to protect the planet, in fighting the complex scientific problems and social quandaries presented by climate change. Youth education represents one of the most effective tools to combat the destructive potential of climate change and cultivate an international understanding among members of the next generation since it is a long-term process that will impact an infinite number of future generations.[2]

The theme of International Youth Day, 2008, was “Youth and Climate change: Time for action.” In his address, Ban Ki-moon, Secretary-General of the United Nations said young people who are adept at spreading new habits and technologies are well placed to contribute to the fight against climate change. Mr. Ban stressed: “They (youth) are adaptable and can quickly make low-carbon lifestyles and career choices a part of their daily lives. Youth should therefore be given a chance to take an active part in the decision-making of local, national and global levels. They can actively support initiatives that will lead to the passage of far-reaching legislation.”[3] A more defined role should be given to the youth to prevent the impact of climate change. It is essential to conduct major studies among youth regarding awareness about climate change as well as role of youth in combating climate change.
